# Estimating Constraints for Protection Factors from HDX-MS Data

**DOI:** 10.1016/j.bpj.2019.02.024

**Published:** 2019-03-05

**Authors:** Simon P. Skinner, Gael Radou, Roman Tuma, Jeanine J. Houwing-Duistermaat, Emanuele Paci

**Affiliations:** 1 Astbury Centre for Structural Molecular Biology, University of Leeds, Leeds, United Kingdom; 2 School of Cellular and Molecular Biology, University of Leeds, Leeds, United Kingdom; 3 Faculty of Science, University of South Bohemia, České Budějovice, Czech Republic; 4 School of Mathematics, University of Leeds, Leeds, United Kingdom; 5 Department of Biostatistics and Research Support, Julius Center, UMC Utrecht, The Netherlands

## Abstract

Hydrogen/deuterium exchange monitored by mass spectrometry is a promising technique for rapidly fingerprinting structural and dynamical properties of proteins. The time-dependent change in the mass of any fragment of the polypeptide chain depends uniquely on the rate of exchange of its amide hydrogens, but determining the latter from the former is generally not possible. Here, we show that, if time-resolved measurements are available for a number of overlapping peptides that cover the whole sequence, rate constants for each amide hydrogen exchange (or equivalently, their protection factors) may be extracted and the uniqueness of the solutions obtained depending on the degree of peptide overlap. However, in most cases, the solution is not unique, and multiple alternatives must be considered. We provide a statistical method that clusters the solutions to further reduce their number. Such analysis always provides meaningful constraints on protection factors and can be used in situations in which obtaining more refined experimental data is impractical. It also provides a systematic way to improve data collection strategies to obtain unambiguous information at single-residue level (e.g., for assessing protein structure predictions at atomistic level).

## Introduction

Dating back to the pioneering work of Linderstrøm-Lang (1), the spontaneous exchange of the amide hydrogens of a protein with deuterium from solvent containing deuterium oxide (^2^H_2_O) has been extensively used to investigate protein folding (2, 3, 4, 5). The key to interpreting hydrogen/deuterium exchange (HDX) kinetics is the fact that exchange occurs faster for amides that are solvent exposed and/or not involved in hydrogen bonds.

For small proteins, site-specific deuterium incorporation can be measured using NMR (6); for larger proteins and assemblies, detection of hydrogen-deuterium exchange by high-resolution mass spectrometry (MS) has been established as a viable alternative (7, 8, 9, 10). HDX-MS relies on the measurable difference of mass between the deuterated and nondeuterated polypeptide chains. To obtain more specific information, the polypeptide is further fragmentated by proteolysis at a low pH and a low temperature (i.e., conditions that reduce exchange and preserve the isotopic pattern during analysis (11, 12)). This allows measurement of exchange for specific fragments of the polypeptide chain (usually covering 10–20 amino acids) (13). Under MS analysis conditions, deuterium incorporated into exchangeable side chain groups and N-terminal amines is rapidly back exchanged, and as a consequence, HDX-MS is only sensitive to the backbone amide exchange.

HDX-MS yields the overall mass change over time for a whole peptide fragment (usually presented as a centroid of the isotopic envelope) but does not provide direct information on the exchange rate of individual residues. The ratio of the observed exchange rate to the maximal one (i.e., that measured for a completely unstructured peptide) is the so-called protection factor. Protection factors contain both structural and dynamic information; the degree of protection of amide hydrogen from solvent deuterium correlates to the degree of involvement in secondary and tertiary structure (14).

Monitoring the incorporation of deuterium for each peptide fragment over time yields exchange kinetics; this contains information about local and global stability averaged over all amide NH groups within the peptide. Such HDX-MS data are usually limited to qualitative analysis (e.g., by mapping the apparent averaged rate of exchange of different peptides on the available structure and comparing the kinetics of the same fragments under different conditions) (12, 15, 16). One way to achieve single amide resolution is to obtain fragments that differ by exactly one amino acid and calculating the mass difference. Using enzymatic digestion, this is only achievable for a few amino acids under favorable conditions, but recent advances in gas-phase fragmentation (10) (e.g., electron capture dissociation (17) and electron transfer dissociation (18, 19)) suggest that HDX-MS can, in principle, be used to measure hydrogen exchange at single-residue resolution. However, even then, gas-phase scrambling (i.e., rapid migration of the incorporated deuterium among backbone and side chains) still needs to be minimized by carefully optimizing fragmentation and ion source conditions for different peptides (20, 21). This is time consuming and becomes impractical for automated and high-throughput approaches that may be required (e.g., for fold profiling).

A different strategy, pioneered in Englander’s laboratory, exploits information encoded in isotopic envelopes instead of just centroid values (22). More recently, it has been shown that combining isotope envelopes with kinetic information over a wider time window provides single amide exchange rates of a 100 amino acid protein, such as cytochrome *c* (23). Thus, most methods are targeted toward structural use and delineate exchange at individual amino acids while using high quality data with optimized coverage and fragmentation, wide time windows, and resolved isotopic envelopes (24, 25, 26). However, most of the available HDX-MS data are usually in the form of centroid values over time, and the coverage and fragment patterns are far from optimal, limiting the use of such methods on most available data sets. The required uniform coverage and resolution of isotopic envelopes is also hard to achieve for larger proteins and multiprotein assemblies (19). But such data still offer valuable information that can be used to characterize structure and dynamics in cases in which other methods are not viable. Hence, there is a need for a robust method that would be able to extract site-specific protection factors or at least obtain constraints on their range, irrespective of the input data quality.

In this article, we present a statistical method that extracts individual protection factors from HDX-MS measurements of centroid mass variation kinetics obtained for overlapping proteolytic fragments of the polypeptide chain. The method is general, but the degeneracy of the solution (i.e., a set of protection factors for each exchangeable amide hydrogen) depends on the number of peptides and overlap (the more the better), on their length (the shorter the better), and on the range of times at which the measurement has been measured (the broader the better). The accuracy of the predicted protection factors also depends, obviously, on the accuracy of the measurement itself, although we show here that self-consistent use of the data for overlapping peptides also provides a tool to appraise possible experimental errors in the measurement of the deuterium uptake. We demonstrate that, even in the absence of full, redundant fragment coverage of the protein sequence and with measurements performed in a relative narrow time window (10–10^4^ s), the approach provides a relatively small number of solutions, in which for a subset of residues, the protection factors are uniquely determined, whereas for others, discrete sets of values are possible. We included an option to use isotopic envelopes, if available, to further reduce the number of possible solutions and uniquely determine the protection factor of each residue in cases of insufficient information from centroids alone. These features make the method suitable for analyzing a wealth of existing HDX-MS data and extract crucial information at single-residue level. The method also quantifies, in a statistically rigorous manner, the information contained in the data. When the information in the HDX-MS data provides multiple answers, the tool can be used to guide further experiments that effectively resolve the remaining ambiguity.

## Methods

### Principles of hydrogen-deuterium exchange probed by MS

At a neutral pH, the exchange is fast for solvent-exposed amides, whereas hydrogen bonding (e.g., within helices or *β*-sheets) slows it down. When fully exposed, the exchange of the amide follows first-order kinetics with (intrinsic) rate *k*_*int*_, which depends on the temperature, solution pH, and side chains of the two neighboring residues (27). Within a folded protein, the exchange of amide hydrogen requires local “opening” of the structure and can be approximated as a two-step process (28):

(1) $$NH_{cl}\overset{k_{cl}/k_{op}}{↔}NH_{op}\overset{k_{int}}{\to }ND_{op},$$

where *k*_*cl*_ and *k*_*op*_ are the local “closing” and “opening” rates. The observed deuterium uptake rate, *k*_*obs*_, can be expressed as

(2) $$k_{obs}=\frac{k_{int}k_{op}}{k_{int}+k_{op}+k_{cl}}.$$

Two limiting regimes, usually referred to as EX1 and EX2, are invoked in interpreting HDX kinetics of proteins. For both regimes, the protein is considered to be in native conditions (i.e., $k_{cl}\gg k_{op}$). In the EX1 limit, $k_{int}\gg k_{cl}$ implies that the amide exchanges as soon as it becomes exposed to solvent (i.e., *k*_*obs*_ = *k*_*op*_). In this regime, the exchange is limited by slow conformational changes that are usually associated with global unfolding (29) or cooperative changes in quaternary structure (16). This regime is readily discerned by a bimodal pattern of isotopic distribution in mass spectra (undeuterated and deuterated species) and by pH independence. In the EX2 limit, $k_{cl}\gg k_{int}$,

(3) $$k_{obs}=\frac{k_{int}}{P},$$

where *P* = *k*_*cl*_/*k*_*op*_ is a protection factor for the particular amide hydrogen. The EX2 limit governs exchange under native conditions and is sensitive to local stability. In the EX2 regime, the kinetics is sensitive to pH (through *k*_*int*_), and the corresponding isotopic envelope moves progressively to the fully deuterated limit.

HDX-MS measures the change in mass upon deuteration of proteolytic fragments of the polypeptide chain. The deuterium uptake *D*_*j*_ for peptide *j*, starting at residue *m*_*j*_ and *n*_*j*_ residues long, is

(4) $$D_{j}\left(t_{k},\left\{P_{i}\right\}\right)=\frac{1}{n_{j}}\sum_{i=m_{j}+1}^{m_{j}+n_{j}-1}\left(1-e^{-\frac{k_{i}^{int}}{P_{i}}t_{k}}\right),$$

where *P*_*i*_ is the protection factor of residue *i* and *t*_*k*_ is a set of time points (the sum ignores the first residue of the fragment because it becomes amine during proteolysis and back exchanges rapidly during analysis). A rapid back exchange is sometimes reported for the second residue (30). However, loss of deuterium at this site is usually considered as a part of correction for overall back exchange. To account for the site-specific differences in back exchange, the exact conditions of analysis, including digestion time and duration of all high-performance liquid chromatography steps, would have to be considered, and this information is seldom available in the necessary detail. Hence, for the sake of wide applicability and simplicity, we forgo this exact approach at the expense of overall accuracy.

### Determination of protection factors from HDX-MS data

The task can be reformulated as determining the set {*P*_*i*_} so that

(5) $$D_{j}^{pred}\left(t_{k}\right)=D_{j}^{exp}\left(t_{k}\right)+\varepsilon_{j,k},$$

for each *j* and *k*, where *ε*_*j,k*_ is the deviation between experimental data and model.

This problem corresponds to that of determining the set (or sets) of protection factors {*P*_*i*_} corresponding to the minimum of the cost function,

(6) $$C\left(t_{k},\left\{P_{i}\right\}\right)=\sum_{j}\sum_{k}w_{jk}{\left[D_{j}^{pred}\left(t_{k},\left\{P_{i}\right\}\right)-D_{j}^{exp}\left(t_{k}\right)\right]}^{2}=\sum_{j}\sum_{k}{\left(\varepsilon_{j,k}\right)}^{2},$$

where *w*_*jk*_ is a weight; if an average of $D_{j}^{exp}\left(t_{k}\right)$ over repeated measurements is available, an appropriate choice of the weight would be the inverse of the SD. The right-hand term originates from the fact that each measured value *D*^*exp*^ is affected by an experimental uncertainty, which is often not reported in the literature. Hence, the minimum of the cost function *C* is generally not zero. Even in the absence of experimental error, the solution (i.e., sets of protection factors for which *C* = 0) is, in general, not unique. For example, if the deuterium uptake of a single peptide is available that is long relative to the number of time points at which the experiment has been performed, an infinite number of sets of protection factors that minimize *C* exist. However, if measurements are available for contiguous overlapping peptides along the polypeptide chain, a unique or a finite number of sets of protection factors for which *C* is close to its global minimum can be determined for each residue occurring in the contiguous region. The existence and the possible uniqueness of the solution strongly depend on the set of experimental data.

To determine sets of protection factors compatible with the experimental deuterium uptakes, we opted to perform a random search a number of times (e.g., 10^4^) and use the set with the lowest *C* as an initial condition for a least-squares minimization. In the “Clustering” section below, we detail how convergence of the random search can be assessed. A sequential quadratic programming approach, as implemented in SciPy 0.19.1 (31), was used to minimize the cost function.

### Clustering

For each residue, the median and the interquartile range of the predicted ln(*P*) were computed; for residues with large interquartile range, histograms of ln(*P*) were plotted. The distributions often appear to be multimodal, and the solutions for neighboring residues within the same peptide or overlapping peptides are, in principle, correlated. Hence, we applied a model-based clustering method (32) to obtain sets of ln(*P*) for each region (of length *L*) of the polypeptide chain that is continuously covered by resolved peptides. Let *Λ* be the number of clusters (i.e., the number of solutions for a region). To estimate the solutions $\mu_{\lambda }=\left(\begin{array}{c} \mu_{\lambda 1}\\ \vdots \\ \mu_{\lambda L} \end{array}\right)\;\lambda =1..\Lambda$, the following likelihood was maximized:

(7) $$L\left(\pi_{1},..,\pi_{\Lambda },\mu_{1}\ldots \mu_{\Lambda },\Sigma_{1}\ldots \Sigma_{\Lambda }|\left\{P\right\}\right)=\prod_{j=1}^{J}\sum_{\lambda =1}^{\Lambda }\pi_{\lambda }f_{\mu_{\lambda },\Sigma_{\lambda }}\left({\left\{P\right\}}_{j}\right),$$

where *J* is the number of generated data vectors for the considered region,*π*_*λ*_ is the fraction of data vectors belonging to cluster *λ*, and $f_{\mu_{\lambda },\Sigma_{\lambda }}\left(\cdot \right)$ a multivariate normal distribution with mean $\mu_{\lambda }=\left(\begin{array}{c} \mu_{\lambda 1}\\ \vdots \\ \mu_{\lambda L} \end{array}\right)\;$and covariance Σ_*λ*_ (an *L* × *L* matrix with diagonal elements equal to the residue specific variances). The log likelihood was maximized by using an expectation maximization algorithm (33). The number of solutions *Λ* was chosen based on the lowest Bayesian information criteria value. Convergence of the random search of sets of protection factors (see above) is reached when additional solutions do not affect the result of the clustering procedure (i.e., the number of clusters does not increase further). Note that ***μ***_*λ*_ is the unbiased estimate for the vector of protection factor values corresponding to solution *λ*. The generated data support, however, other protection factor values for this solution as well. Specifically, for residue *i*, ***μ***_*λi*_ ± *σ*_*λii*_ is the ∼70% reference interval comprising all these solutions. For simplicity, we write that ***μ***_*λ*_ is solution *λ*.

### Calculation of the isotopic envelope

**Figure 1:**
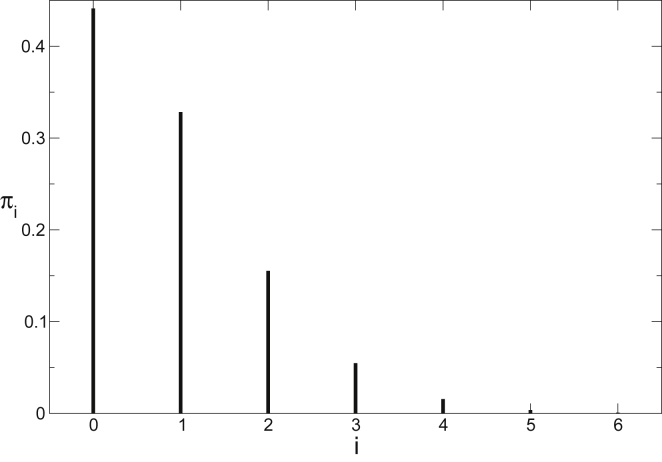
Isotopic envelope for the undeuterated form of the 12-residue peptide IDSQVLCGAVKW. Lines represent the probability of isotopic variants with isotope number +i relative to the monoisotopic species with m/z = 1318.68.

The isotopic-resolved mass spectrum of a peptide is shown in Fig. 1; the height of each peak is the frequency *π*_*i*_ of natural occurrence of isotopic variant with mass +*i* relative to the monoisotopic species of the undeuterated peptide.

As amide hydrogen exchanges to deuterium, the intensity of each peak changes with probability, depending on the rate of exchange of each amide. For a peptide with *n* exchangeable amides, the probability that *k* (0 ≤ *k* ≤ *n*) have exchanged at time *t* is

(8) $$\Pi \left(k;t\right)=\sum_{\begin{array}{c} A\subset \left\{1,\ldots n\right\}\\ \left|A\right|=k \end{array}}\prod_{i\in A}D_{i}\left(t\right)\prod_{j\in \left\{1,\ldots ,n\right\}\setminus A}\left(1-D_{j}\left(t\right)\right).$$

The isotopic envelope at time *t* is the probability of a species with isotopic number *i* + *k*, which is in turn the joint probability *π*_*i*_*Π*(*k*;*t*).

The algorithms have been implemented using version 3.6 of the Python programming language, using the NumPy, SciPy, and Cython libraries. This code is freely available to academics via a GitHub repository (https://www.github.com). Commercial enterprises can obtain this code subject to a proprietary license.

## Results

### Test case

**Figure 2:**
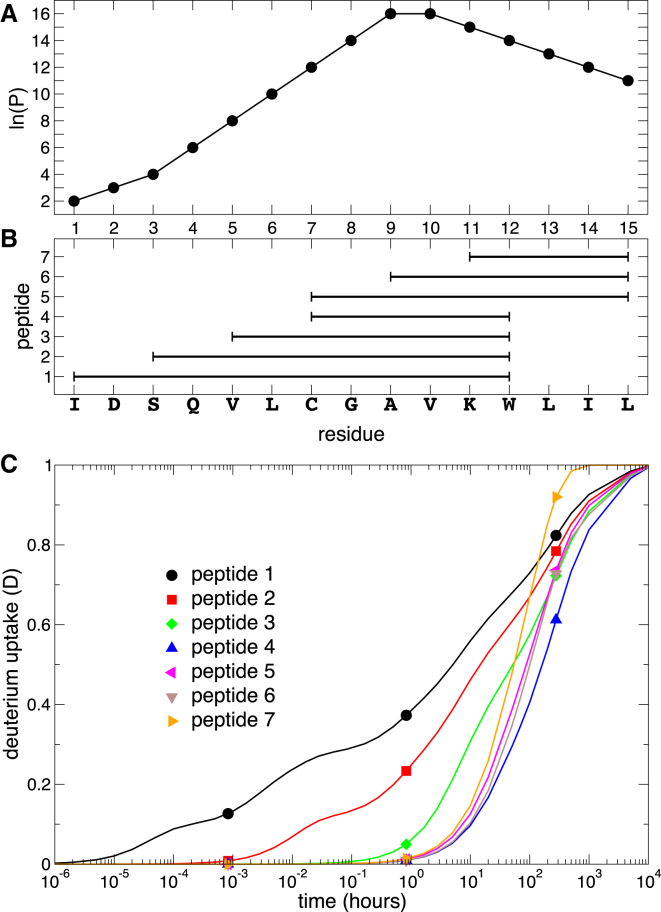
(*A*) Protection factors for a 15-residue sequence, peptide fragments for which the deuterium uptake (*B*) has been calculated using Eq. 4, and intrinsic exchange rates at 300°K and pH 7; (*C*) the three time points (*symbols*) represent all the experimentally available data. To see this figure in color, go online.

We have first tested the approach on simple synthetic data for a 15-residue peptide, generated for an arbitrary set of fragments and times using a fixed set of reference protection factors and Eq. 4 (Fig. 2).

To determine the values ln(*P*) that minimize the cost *C*, we first generate values with a uniform distribution with boundaries 0 ≤ ln(*P*) ≤ 20 (i.e., we assume that the exchange rate of an amide can be as fast as in a completely unstructured peptide and up to 5 × 10^8^ times slower). The process is repeated many times (until convergence is reached; in this case, 5 × 10^3^ times), and the set with the lowest *C* is used as an initial guess for the subsequent minimization (here, by using the sequential least-squares quadratic programming method), with constraints 0 ≤ ln(*P*) ≤ 20. The whole procedure is then repeated many times, and sets of protection factors with lowest *C* are then selected for further analysis. Details are provided in Methods.

**Figure 3:**
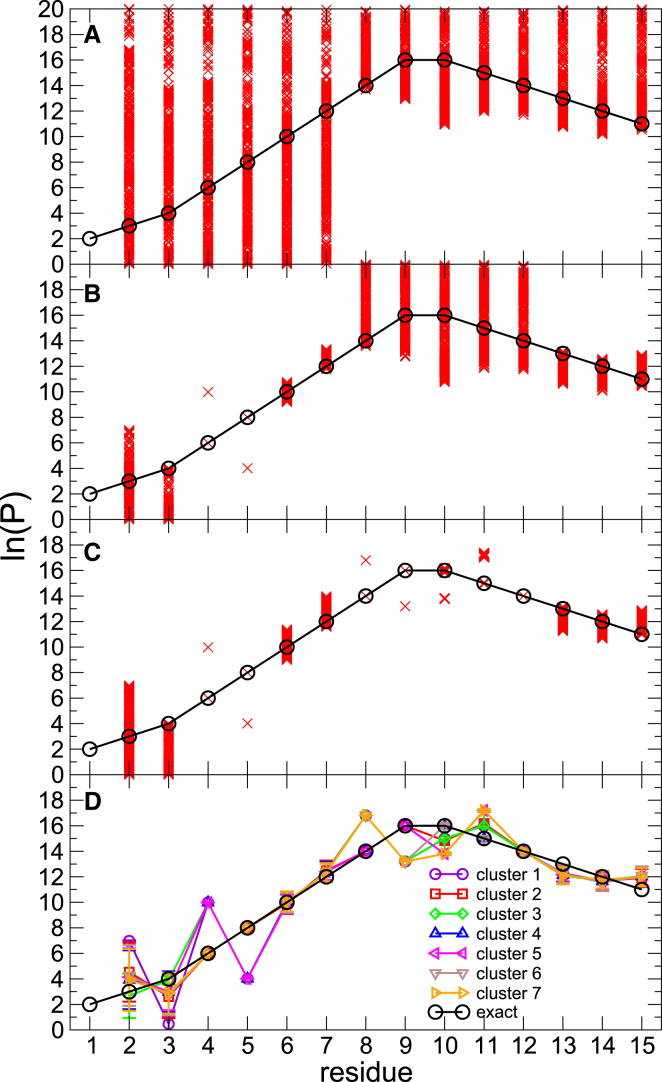
Predicted protection factors in the case in which only deuterium uptake for peptides 1 and 5 are used (*A*), peptides 1–3 and 5 (*B*), and peptides 1–7 (*C*). The reference protection factors from which the reference deuterium uptake has been calculated are shown as black circles. Red crosses represent the predicted protection factor that provides exact agreement between the calculated and reference deuterium uptake. In (*D*) are shown the means of the seven clusters (i.e., a set of means and SDs for each residue) that represent all the possible solutions in (*C*). To see this figure in color, go online.

To illustrate the outcomes, we performed the above procedure using the deuterium uptake for peptides 1 and 5 only, peptides 1–3 and 5, and all peptides 1–7 (Fig. 2 *B*). We used only deuterium uptake at three time points (3 s, 50 min, and 280 h). Even when using only two peptides with a marginal overlap, predictions for each residue in the sequence can be obtained, but as expected, multiple sets of compatible protection factors (with *C* = 0 in the absence of experimental error) were found, and their range is large (Fig. 3 *A*). When simulated data for the partly overlapping peptide sets 1–3, 5, and 1–7 are used, the ambiguity progressively decreases (Fig. 3, *B* and *C*). In all cases, the exact protection factor for each residue is found among the generated solutions. However, for the whole chain, the exact solution is not represented by a single cluster because of the scarcity of overlapping peptides, which in turn does not provide sufficient constraint.

The prediction of the individual protection factor for each residue suggests that the number of possible solutions is infinite for the whole peptide. However, discrete values emerge for some residues as the coverage and overlap increases (e.g., residues 4 and 5 in Figs. 3 *B* and residues 4, 5, 6, and 7 in Fig. 3 *C*, respectively). For residues 4 and 5, the correct values for ln(*P*) are 6 and 8, respectively, and these occur simultaneously in one solution set. Yet another solution with ln(*P*) values 10 and 4 is also possible because these reproduce the same total uptake for the time points considered. However, other combinations of these values are not compatible with the data, and thus for residues 4 and 5, we have only two possible solutions and not four.

The examples given above illustrate a general feature that the estimate of the protection factor for a given residue is linked to those of neighboring residues. As a result, the number of total solutions is considerably smaller when considered simultaneously. A way to estimate the number of plausible solutions and the corresponding sets of protection factors is to use a model-based clustering method as described in Methods. Fig. 3 *D* shows seven different possible sets (*Λ* = 7 as determined by Bayesian information criteria, see Methods) of protection factors; the error bars represent the variance $\Sigma_{\lambda }$ around the mean ***μ***_*λ*_ for each solution *λ* =,…,*Λ*, where variance and mean are vectors of dimension equal to the length of the region (here, 15 residues).

**Figure 4:**
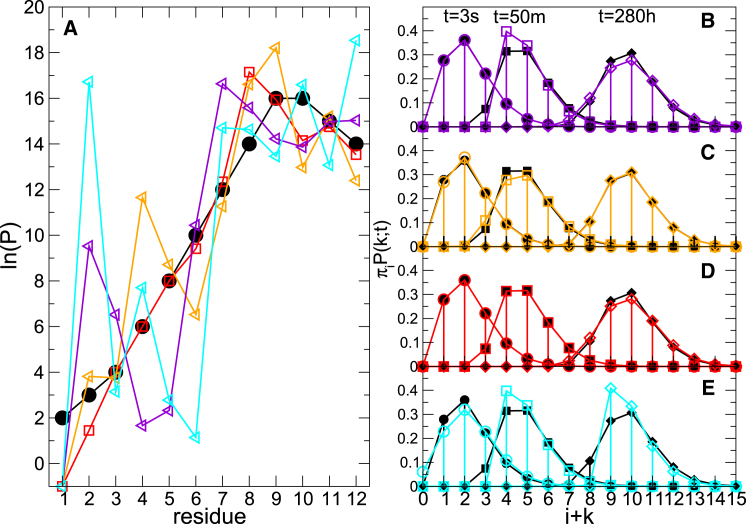
(*A*) Four sets of protection factors satisfying experimental constraints (*color symbols*) and reference protection factors (*black circles*). (*B*–*E*) Isotopic envelopes calculated for the protection factor sets in (*A*) are shown. The sets of protection factors closer to the exact ones (in *orange* and *red*) result in an isotopic envelope (*C* and *D*) closer to the exact one (*black solid symbols*). Circles, squares, and diamonds in (*B*–*E*) are used to indicate different time points. To see this figure in color, go online.

Isotopic envelopes, if available, can be further used to reduce the number of solutions. The alternative patterns of protection factors are first used to generate the deuterium uptake curve for each amino acid and, from Eq. 8, the time evolution of the corresponding isotopic envelope for each peptide. Focusing on the 12-residue peptide 1 (Fig. 2 *B*), four sets of protection factors that exactly satisfy the experimental deuterium uptake are shown in Fig. 4 *A*, together with the reference values from which deuterium uptake has been calculated (here, we use the case in which only peptides 1 and 5 are used in the prediction, and the ambiguity is largest). The isotopic envelopes corresponding to the different sets of protection factors are shown in Fig. 4 *A* for the three different time points at which the deuterium uptake has been calculated. The isotopic envelopes differ subtly. This can be used to discriminate between the generated protection factor sets. The set producing envelopes closest to the exact isotopic envelopes over all considered time points (i.e., the one best representing the modeled “experimental data”) also yields protection factors best matching the exact values. Thus, when well-defined isotopic envelopes are available, such quantitative comparison can help to further discriminate between the alternative sets of protection factors obtained by cluster analysis.

### Application

**Figure 5:**
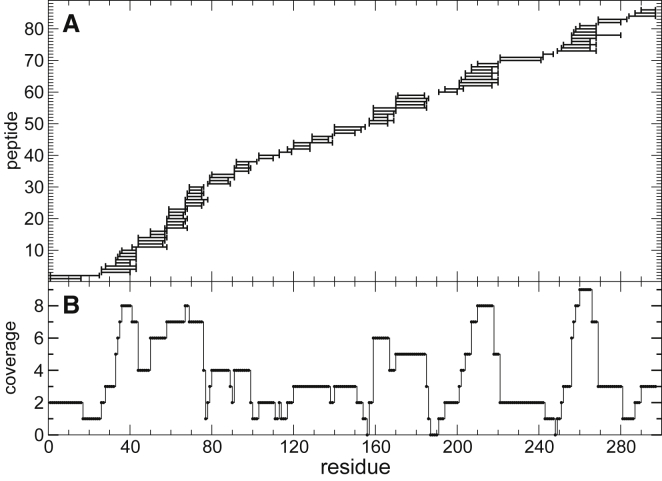
(*A*) Peptides (86 in total) obtained by peptic digestion of the protein C3d studied by HDX-MS by Devaurs et al. (35); fragments range between 6 and 26 amino acids in length. (*B*) Coverage (i.e., number of peptides in which an amino acid occurs) of 297 amino acids (only six amino acids are not covered) and their protection factor are thus undetermined.

As a real-world example of the approach, we use HDX-MS data recently published for the protein, C3d, which is a fragment of the complement component C3 (34). C3d is a single-domain protein composed of 297 residues, in which residue 1 corresponds to residue 991 of the full C3 molecule. C3d contains 12 *α*-helices and five 3_10_ helices that are organized into an *α*-*α* barrel in which most consecutive helices alternate between the inside and the outside of the protein core. Knowledge of the structure, however, is irrelevant and never used in our approach. The protein C3d has been chosen as a test case for the availability of HDX-MS data with high coverage of the sequence (∼98%) and high redundancy (on average, each amino acid is occurring in 3.6 independently probed fragments) (35) (Fig. 5). Measurements of deuterium uptake have been obtained at pD 7.5 and 297°K temperature for seven time points (10, 30, 100, 300, 1000, 3000, and 10,000 s).

**Figure 6:**
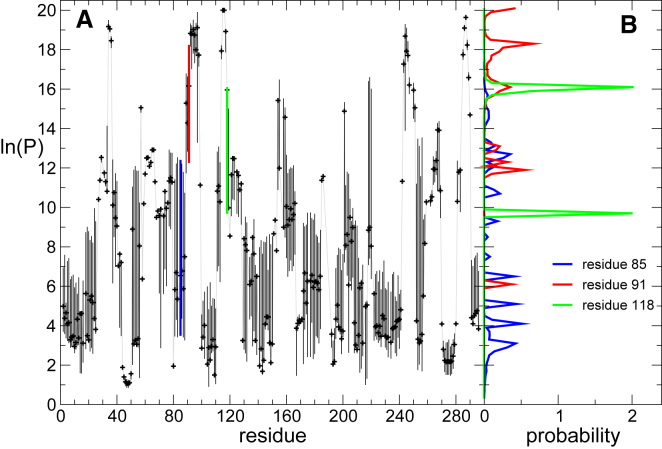
Descriptive of the generated data. (*A*) Median (*black symbols*) and interquartile range (*black bars*) of all **ln**(***P***) values are compatible with the experimental data for all exchangeable amide hydrogen, excluding those not covered by any fragment probed by the HDX-MS measurement (156, 187–190, and 248; see Fig. 5*A*). Values are computed from over 5000 independent minimizations that resulted in a cost ***C*** < 0.006. (*B*) For three positions, the distributions (histograms) of **ln**(***P***) are shown; the histograms show that the predicted **ln**(***P***) follows a multimodal distribution. The bars representing the interquartile range of the three residues are shown in the same color in (*A*). To see this figure in color, go online.

**Figure 7:**
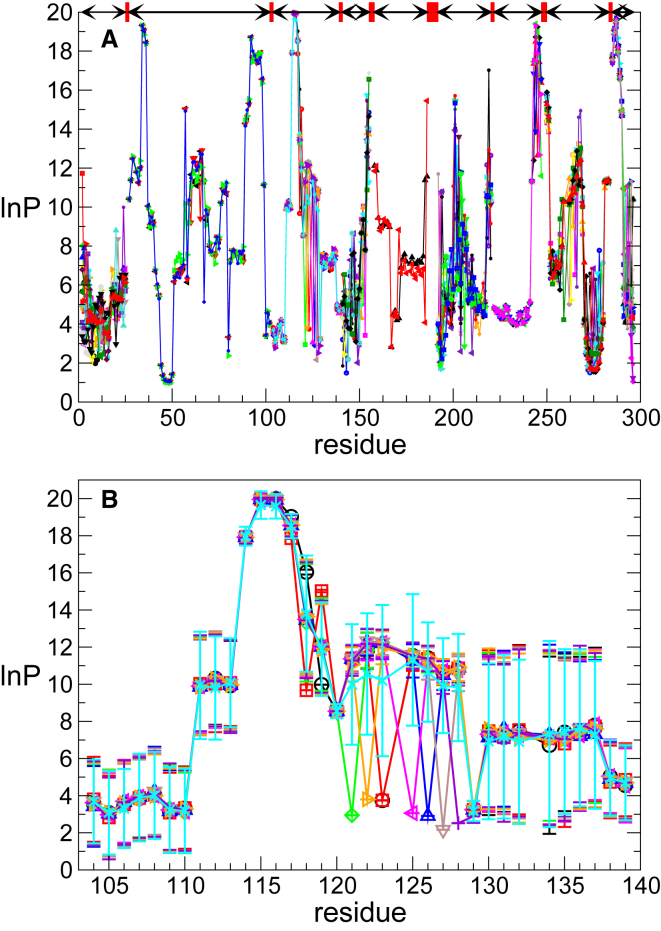
Results of cluster analysis. Sets of estimated multivariate means for **ln**(***P***) are shown. (*A*) Top shows the results for all contiguous regions (i.e., regions of the polypeptide chain covered by overlapping peptides) as arrows with red boxes representing gaps between these regions. Per contiguous region, a colored curve represents one set of multivariate solutions across the residues of this region. The multivariate means for each cluster (represented by a Gaussian) are shown in different colors. Note, fewer colors are used in regions with few well-defined solutions. (*B*) The nine sets of multivariate solutions obtained for the contiguous region encompassing residues 103–139 are shown. Error bars represent 1 SD. To see this figure in color, go online.

Analysis of the sets of ln(*P*) that best fit the sets $D_{j}^{exp}$ is reported in Figs. 6 and 7. In Fig. 6, the median and interquartile ranges are shown. Whereas for some residues, the protection factors are narrowly defined, for others, the interquartile ranges show large variations between values compatible with the experimental data (Fig. 6 *A*). As for the test case above, in most cases, the distributions of the protection factors for such residues cluster narrowly around a discrete set of possible values (illustrated in Fig. 6 *B* for selected residues). The uncertainty is restricted by the constraint that the property of each residue is the same in different overlapping peptides, and here, 98% of the sequence is covered redundantly by numerous peptides (Fig. 5). This is the reason why, for several residues, only a single protection factor is compatible with the data, and discrete sets of values can be found for the rest.

As suggested by Fig. 6 *B*, the empirical distribution of protection factor values can be approximated by a mixture of Gaussian distributions, in which the means of the Gaussian distributions represent the solutions. Because the protection factors for residues within a region with contiguous coverage of overlapping peptides are interdependent, further insight can be obtained by modeling the values of all residues in the region simultaneously (i.e., by using a mixture of multivariate Gaussian distributions). The vector of means of a multivariate Gaussian component represents a solution for the set of the protection factors across the region. In the case presented here, there are nine contiguous regions that have been modeled independently. The multivariate means for all contiguous regions of the polypeptide chain are depicted in Fig. 7 *A*. This illustrates variability in the number of plausible solutions between different regions, depending on the pattern and completeness of coverage. To further explore the dependence of different solutions across the residues, the multivariate means for a single contiguous region (residue 103–139) are shown in Fig. 7 *B*. For some residues, the protection factors are uniquely defined, whereas for others, alternative values are possible but only for a limited number of their combinations. Such a multivariate representation of the solutions also helps to discriminate regions with well-constrained protection factors from those with considerable ambiguity and also gives upper and lower bounds on possible values.

### Comparison with experimental deuterium uptake data

**Figure 8:**
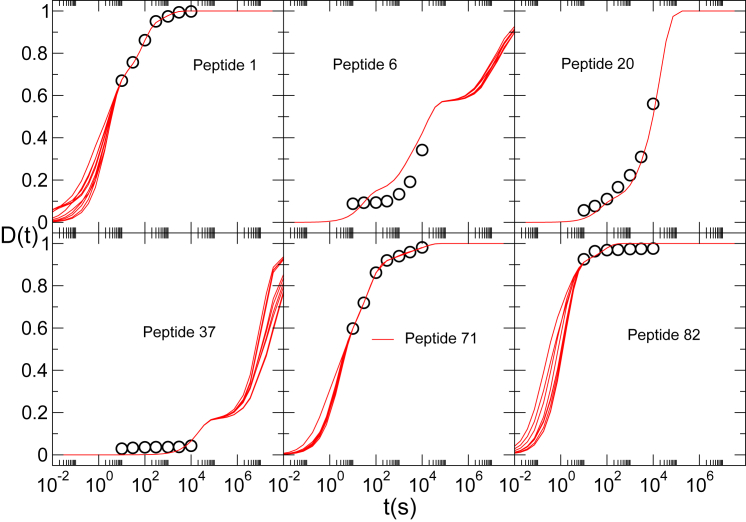
***D***^***exp***^***(t***_***k***_***)*** (*circles*) and ***D***^***pred***^***(t)*** (*continuous lines*) as a function of time for six of the 86 peptides experimentally probed for protein C3d. Continuous lines correspond to the 10 solutions with lowest C. To see this figure in color, go online.

As presented above, multiple sets of protection factors can satisfy the experimental data equally well. The experimentally measured and estimated deuterium uptake kinetics for six different peptides are shown in Fig. 8. Different sets of protection factors fit the experimental data equally well but usually deviate from each other outside the measured time window. For each peptide, the 10 sets of protection factors with the lowest cost (among the ∼5000 sets with cost *C* < 0.006) have been used to calculate the deuterium uptake over a time interval covering 10 orders of magnitude. For peptides 1, 71, and 82, we observe that experimental data is perfectly reproduced in the time interval probed by the experiment, but different sets of predicted protection factors provide alternative profiles for times shorter than those measured experimentally. This is related to the fact that the three peptides fall in a region of low coverage (see Fig. 5) but also highlights the necessity of measurements at shorter times (<1 s). The opposite is true for peptide 37, in which the experimental data only cover times at which the exchange has not yet occurred, and the variability in the predicted protection factors is responsible for a different time dependence of the predicted deuterium uptake at long time (>10^6^ s). The prediction of the deuterium uptake for peptide 20 appears to be robust at all timescales, most likely due to the information contained in the considerable number of peptides that partially overlap with it. The deuterium uptake of peptide 6 represents the most evident case in which the fit is consistently suboptimal, and the possible reasons are discussed below.

**Figure 9:**
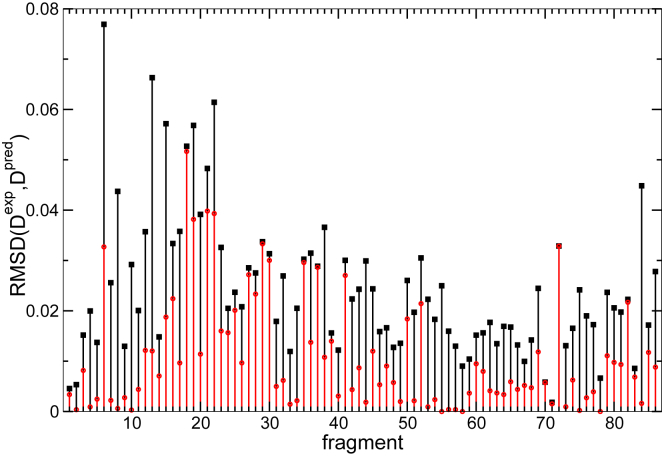
Deviation between experiment and calculation for each peptide. In black is shown the deviation between the model and experimental data when the deuterium uptake of all peptides is optimized simultaneously. In red is shown the same deviation when each peptide is fitted individually; a value different from zero means that no set {***P***_***i***_} can be found that perfectly fit the experimental deuterium uptake, corresponding to a lower limit for the average experimental error for that peptide. Deviation is largest for peptide 6, which is also highlighted in Fig. 6. For most other peptides, the deviation is small and within the presumed statistical error of the experimental measurement. To see this figure in color, go online.

The root mean-square deviation (averaged over all the different sets of predicted protection factors) between experimental $D_{J}^{exp}\left(t_{k}\right)$ and calculated *D*_*J*_(*t*_*k*_) is shown in Fig. 9. Also shown is the deviation between experiment and model when the cost function is minimized for individual peptides (in which case, the protection factors of individual residues are severely underdetermined). A value larger than zero highlights that the experimental deuterium uptake cannot be fitted exactly with the sum of exponentials in Eq. 4; this provides a lower limit of the experimental error in the measurement or else may be an indication that some of the assumptions, such as that the protection factor of each residue is uniquely defined (which is not the case if populations of different conformers exist) or that exchange occurs in the EX2 regime, are not satisfied. Most peptides can be fitted exactly individually but not when considered simultaneously (this is, for example, the case of the highly overlapping peptides 56–59 that all include residues 171–183) because of experimental errors associated with the deuterium uptake for the peptide and/or those partially overlapping and flanking peptides.

## Discussion

Many HDX-MS studies report results for a set of peptides obtained by enzymatic fragmentation of the protein. Results are most often reported as deuterium uptake curves (measured as centroid mass) or “butterfly charts” and thus provide only qualitative information at the resolution level of several amino acids. Multiple successful methods to extract rates as a single-residue level have been developed (23, 24, 25, 26, 36) using data with optimized fragmentation patterns.

In this article, we have presented a method that yields estimates or constraints for HDX protection factors at the levels of individual residues from HDX-MS centroid mass measurements as a function of time. Our approach provides alternative sets of protection factors that agree with the experiment. For some residues, the prediction provides a set of unique protection factor values. For others, we obtain a range of solutions that, in most cases, is well represented by a relatively small set of discrete values. These considerations depend strongly on the set of experimental data. The existence of a unique or of finite sets of solutions depends on the length and overlap of the peptide fragments. Also, peptides that exchange much faster than ∼10 s or slower than 10^4^ s, which is the interval usually probed, provide little or no information.

The contribution of statistical and systematic errors to the experimental data deserves further discussion. Statistical errors could be easily included in this approach by accepting all the solutions that satisfy the experimental data within the error (for example, by weighing the contribution from the deuterium uptake of each peptide by a factor inversely proportional to the SD over multiple measurements). Systematic errors depend mostly on the correction for back (and sometimes, also forward) exchange. A phenomenological correction for back exchange could be included in this approach by minimizing the function $C\left(\text{ln}\left(P\right),\alpha \right)=\sum_{j}\sum_{k}{\left[\left(1-\alpha_{j}\right)D_{j}\left(t_{k}\right)-D_{j}^{exp}\left(t_{k}\right)\right]}^{2}$ where *α*_*J*_ is the fraction of deuterated amides that exchanges back to hydrogen for a fragment *j* (i.e., assuming that the ratio of exchange and back-exchange events is constant over all time points).

To demonstrate the workings of our method, we used a data set in which fragments redundantly cover most of the protein sequence. The result is not a single set of protection factors but a family of them. This is inevitable when fragments are long and coverage of the sequence uneven. In contrast to the previous methods (26, 36), we aim to determine all the sets of exchange rates (or equivalently protection factors) for each residue compatible with the experimental data and reduce the underdetermination of the problem by exploiting the interdependence of the solution at different sites. The method proposed here represents a search for self-consistent solutions to the problem, followed by a statistical analysis of these solutions, yielding the best estimates and corresponding uncertainty of the protection factor values that reproduce the deuterium uptake measured experimentally. For some residues, it is possible to estimate unique protection factors within uncertainly intervals. For others, the distribution of the protection factors is multimodal, with plausible values distributed narrowly around a discrete set of means. A multivariate cluster analysis provides the best estimate of the protection factors for adjacent residues because the value for one residue depends on those of others within the same or in overlapping peptides. The approach primarily uses centroid data, which represents the vast majority of the published HDX-MS results; hence, it is widely applicable. The ambiguity of the estimated protection factors depends primarily on the redundancy of the coverage of the sequence provided by the experimental data set. Availability of isotopic envelopes further reduces this ambiguity.

The method proposed here also gives an indication of how the uncertainty could be reduced. For example, it highlights regions of the polypeptide chain, where a different fragmentation approach would provide additional constraints and reduce the uncertainty. Where the time evolution of the isotopic envelope is available for specific peptides, it provides a practical way to deconvolute the spectra and reduce or eliminate the uncertainty on the estimation of the protection factors of the peptide and reduce that of contiguous peptides. Given the increased availability of mass spectrometers and the automation of the exchange and fragmentation process, the method presented here has strong potential to turn a mostly qualitative analysis into a quantitative one.

Protection factors for each residue can be converted structural information (37, 38, 39, 40) and used to generate or validate models of proteins (5, 41). Although deuterium uptake alone can be used to validate structural models (42), the information contained in protection factors provides a much more stringent validation tool.

## Author Contributions

S.P.S., R.T., E.P., G.R., and J.J.H.-D. designed and performed the research. S.P.S., E.P., and J.J.H.-D. analyzed the data. S.P.S., R.T., E.P., and J.J.H.-D. wrote the article.
